# Salivary bacterial shifts in oral leukoplakia resemble the dysbiotic oral cancer bacteriome

**DOI:** 10.1080/20002297.2020.1857998

**Published:** 2020-12-09

**Authors:** Divya Gopinath, Rohit Kunnath Menon, Chong Chun Wie, Moinak Banerjee, Swagatika Panda, Deviprasad Mandal, Paresh Kumar Behera, Susanta Roychoudhury, Supriya Kheur, Michael George Botelho, Newell W. Johnson

**Affiliations:** a Faculty of Dentistry, University of Hong Kong, Hong Kong SAR, China; b Oral Diagnostics and Surgical Science, School of Dentistry, International Medical University, Kuala Lumpur, Malaysia; c Clinical Dentistry Division, School of Dentistry, International Medical University, Kuala Lumpur, Malaysia; d School of Pharmacy, Monash University, Selangor, Malaysia; e Human Molecular Genetics Lab, Rajiv Gandhi Centre for Biotechnology, Trivandrum, India; f Department of Oral Pathology and Microbiology, Siksha O Anusandhan University, Bhubaneswar, India; g Head and Neck Oncology, Acharya Harihara Regional Cancer Centre, Bhubaneswar, India; h Basic research, Saroj Gupta Cancer Centre and Research Institute, Kolkata, India; i Department of Oral Pathology and Microbiology, D.Y. Patil Dental College, Pune, India; j Menzies Health Institute Queensland and School of Dentistry and Oral Health, Griffith University, Australia; k Faculty of Dentistry, Oral and Craniofacial Sciences, King’s College London, UK

**Keywords:** Saliva, bacteriome, oral microbiome, leukoplakia, oral cancer

## Abstract

**Objective**: While some oral carcinomas appear to arise *de novo*, others develop within long-standing conditions of the oral cavity that have malignant potential, now known as oral potentially malignant disorders (OPMDs). The oral bacteriome associated with OPMD has been studied to a lesser extent than that associated with oral cancer. To characterize the association in detail we compared the bacteriome in whole mouth fluid (WMF) in patients with oral leukoplakia, oral cancer and healthy controls.

**Methods**: WMF bacteriome from 20 leukoplakia patients, 31 patients with oral cancer and 23 healthy controls were profiled using the Illumina MiSeq platform. Sequencing reads were processed using DADA2, and taxonomical classification was performed using the phylogenetic placement method. Sparse Partial Least Squares Regression Discriminant Analysis model was used to identify bacterial taxa that best discriminate the studied groups.

**Results**: We found considerable overlap between the WMF bacteriome of leukoplakia and oral cancer while a clearer separation between healthy controls and the former two disorders was observed. Specifically, the separation was attributed to 14 taxa belonging to the genera *Megaspheara, unclassified enterobacteria, Prevotella, Porphyromonas, Rothia* and *Salmonella, Streptococcus, and Fusobacterium*. The most discriminative bacterial genera between leukoplakia and oral cancer were *Megasphaera, unclassified Enterobacteriae, Salmonella and Prevotella.*

**Conclusion**: Oral bacteria may play a role in the early stages of oral carcinogenesis as a dysbiotic bacteriome is associated with oral leukoplakia and this resembles that of oral cancer more than healthy controls. Our findings may have implications for developing oral cancer prevention strategies targeting early microbial drivers of oral carcinogenesis.

## Introduction

Oral potentially malignant disorders (OPMD) include conditions which harbour an increased risk of transformation to oral cancer when compared to patients with an apparently normal oral mucosa. Oral leukoplakia (LKP) is an OPMD defined as a white plaque of questionable risk having excluded (other) known diseases or disorders that carry no increased risk for cancer. The rates of malignant transformation of LKP vary according to the population as well as histopathologic grades of dysplasia historically categorized into mild, moderate, and severe [1,2]. Histopathologic grading of dysplasia remains the most useful predictor of the malignant transformation potential of LKP [3]. Early detection of oral cancer can help to save lives, lessen the burden of morbidity owing to the more radical surgical resection and/or debilitating chemo/radiotherapy required to manage late-stage cancer, and reduces the economic burden of treatment. Hence, over the years, researchers have explored various methods in the hope of developing sensitive and specific biomarkers for early detection of oral cancer or recognition of an OPMD with a relatively high likelihood of malignant transformation. Unfortunately, no single marker has yet been shown to have sufficient utility to successfully translate to the clinical setting [4].

The oral cavity is the gateway to the gut and dysbiosis in the bacterial communities of the oral cavity have been observed in many human disorders. The advent of next-generation sequencing (NGS) has enabled the parallel identification of the multiple organisms present in a biological sample. Recently there has been a surge of studies that have used NGS to study the bacteriome associated with oral cancer and these have identified significant differences in the relative abundances of bacteria in cancer patients compared to healthy individuals [5–7] and oral bacteriome is currently been explored as a potential biomarker for risk of oral cancer [7]. If particular microbiota were found to promote any aspect of the disease process, this would open the way to antimicrobial treatments as part of cancer therapy.

Given the ease and non-invasive nature of collection, the bacteriome of saliva, or what we choose to call ‘whole mouth fluid (WMF)’ in recognition that it contains gingival crevicular fluid, mucosal transudates and serum exudates because inflammation is invariably present – is currently been explored for its potential to detect disease in a several body systems [4,8]. Several studies have identified particular bacterial taxa in WMF which, by association, may have a role in oral carcinogenesis [9,10]. However, the WMF bacteriome associated with oral leukoplakia is not defined yet. We performed 16S rRNA gene amplicon sequencing to identify bacterial signatures that have the potential to help us understand the risk of malignant transformation, and pathogenesis. In this current study, for the first time in relation to the oral microbiome, we have used the phylogenetic tree placement method to study oral bacteriome, which utilizes a consensus genome constructed with all genomes shared by members of the same clade originating from each node on the phylogenetic tree [11].

## Patients and methods

This study was approved by DY Patil Dental College Hospital, Pune, Maharashtra (DYPY/EC/74/17) and Acharya Harihar Regional Cancer Centre, Cuttack, Orissa (068-IEC-AHRCC), and University of Hong Kong (UW 17–242) Ethics Review Boards. Written informed consent was obtained from all participants and all methods in this study were performed following the relevant guidelines of the Declaration of Helsinki on biomedical research involving human subjects. Seventy-four individuals were included in this cross-sectional study. Subjects were recruited from two institutions: DY Patil Dental College Hospital, Pune, and Acharya Harihar Regional Cancer Centre, Cuttack. Detailed demographic details of the subjects are presented in supplementary table 1. The first group of study subjects consisted of patients with histopathologically confirmed oral squamous cell carcinoma (International Classification of Diseases, 10th revision [ICD-10], codes C02–C06). These sites were chosen as they belong to homogenous paradigm of oral cancer, whereas the rest of the codes belongs to sites which have distinct clinical and molecular signatures [12]. The second group of subjects were patients with oral LKP with moderate or severe dysplasia, confirmed by two oral pathologists using WHO criteria [3]. Only cases with moderate-to-severe dysplasia was chosen so as to ensure the presence of dysplasia and to reduce bias owing to subjectivity in reporting of mild dysplasia. Healthy subjects reporting to these institutions for the removal of asymptomatic third molars were utilized as controls and were matched for age and gender. All subjects exposed to antibiotic therapy within 1 month prior to sample collection, as well as those with a co-existing debilitating illness, were excluded.

Unstimulated WMF was collected from controls and patients after clinical diagnosis of oral cancer or leukoplakia, but prior to any biopsy, surgery, radiotherapy, or chemotherapy. Each subject was asked to refrain from smoking, drinking, or eating for at least 30 min before sample collection. Samples were collected by GeneFix^TM^ Saliva DNA Collection device-1 ml (Isohelix, UK). Patients were asked to spit into the collection device that comes prefilled with stabilization buffer and a funnel for ease of collection. After collection of 1 ml WMF, the device was stored at room temperature. However, the final inclusion of the sample into the study was only after histopathologic confirmation of LKP with moderate or severe epithelial dysplasia or squamous cell carcinoma. The stored samples were shipped at room temperature to Rajiv Gandhi Centre for Biotechnology, Kerala, India, for processing.

### Sample processing and sequencing

The DNA extraction was carried out according to manufacturer instructions using the Gene Fix Saliva Prep 2 Isolation kit (Isohelix, UK) which was specifically optimized for the GeneFix^TM^ Saliva DNA Collectors. Prior to Proteinase K treatment, samples were subjected to 30 minutes of enzymatic lysis at 37°C with lysis buffer containing lysozyme (20 mg/ml) (Sigma-Aldrich, Dorset, UK). The extracted DNA was further stored at −20°C. Amplification of bacterial DNA was performed using PCR primers targeting the 16S rRNA gene V3-V4 (319 F-806 R) and the products were purified with AmpureXPbeads (AGENCOURT). After quantification by real-time quantitative PCR (RT-qPCR – EvaGreenTM), the qualified libraries were sequenced on the Illumina MiSeq System using the PE300 reagent Kit.

### Bioinformatics and statistical analysis

A total of 2,862,675 raw sequences were obtained from MiSeq Illumina sequencing. The raw paired-end sequences were joined into contig, trimmed, and quality filtered using DADA2 [13]. The final dataset contained an average of 29450 sequences per sample. The dataset was subsequently imported into PAPRICA pipeline for sequence binning and to infer taxonomic classification for further processing [12]. Briefly, the phylotype and gene inference analyses were performed by first aligning the quality-controlled query reads to the reference alignment with Infernal, then placing them on the phylogenetic reference tree with pplacer [14]. Separately, taxonomical classification and gene inferences were inferred based on edge placement and consensus identity with either internal or terminal nodes. It is noteworthy that this approach uses ‘phylogenetic placement’ instead of ‘operational taxonomic units’ and hence provides a more intuitive relatedness between taxa using their position in reference to the ‘guide tree’ rather than artificially selected sequence homology [12]. To the best of our knowledge, this is the first use of this approach for the study of oral microbial communities. The sequences were binned into a total of 1646 edges based on PAPRICA. To prevent potential noise, edges with <1000 reads were filtered, and the final abundance table consisted of a total of 283 edges or an average of 73 edges per sample.

The abundance table obtained was next imported into Phyloseq [15] for alpha and beta diversity comparison. Specifically, alpha diversity indices, Shannon, Simpson, and Pielou’s evenness were used to compare the species richness and evenness across samples. On the other hand, the beta diversity or community overlap was derived using permutational multivariate analysis of variance (PERMANOVA), and sparse partial least squares discriminant analysis (sPLS-DA) implemented in the mixOmics R package [16]. sPLS-DA is an extension of the partial least square discriminant analysis (PLSDA). The conventional PLS-DA fitted the loading vectors into different orthogonal dimensions based on the loading weight of the feature in the partial least square regression model [17]. Building on the PLS-DA approach, sPLS-DA included the sparsity assumption in which only a small number of features are responsible in driving a biological event. As such, a LASSO penalization is included for feature selection. This optimized the model by removing the ‘noise’ and increase the prediction accuracy [18]. In this study, the sparsity parameter was determined using the ‘tune.splsda’ function (mixOmics R) that performed fivefold cross-validation. The accuracy of the model was evaluated using the area under the receiver operating characteristic (AUROC) curve. The relationship among the selected features, and with the disease groups was inferred using network analysis. Briefly, a network matrix was produced using the ‘network’ function, and the data were exported into cytoscape ver 3.8.1 to construct the network plot. This report conforms, where applicable, to the STROBE guidelines (Strengthening the Reporting of Observational Studies in Epidemiology).

## Results

### Bacteriome diversity and compositional profile of three groups

**Figure 1.**
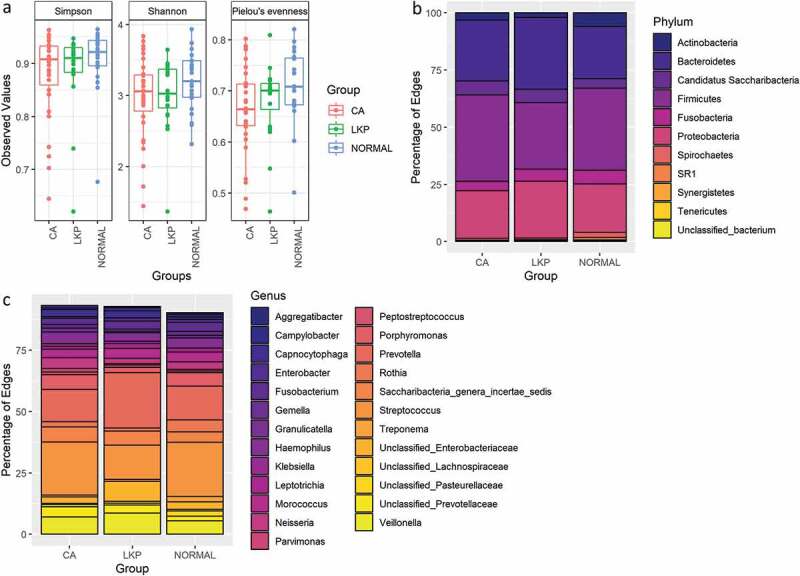
(a) Box plot illustrating microbial diversities between the three groups. No difference in alpha diversity was detected between the groups. (b) Distribution of bacterial communities across three groups at the phylum level. (c) Distribution of bacterial communities across three groups at the genus level

We plotted alpha diversity in samples from each patient group when compared with that found in the other patient groups using various metrics. No significant differences in richness (Shannon and Simpson Diversity Indices) and in evenness were detected (Figure 1(a)). The bacterial compositional differences have been illustrated at phylum and genus levels (Figure 1(b,c)). At the phylum level, *Firmicute*s were the most abundant phyla in the oral cancer group (CA) as well as in controls, whereas *Bacteriodetes* were more predominant in oral leukoplakia (LKP) samples (Figure 1(c)).

### Sparse partial least squares regression discriminant analysis

**Figure 2.**
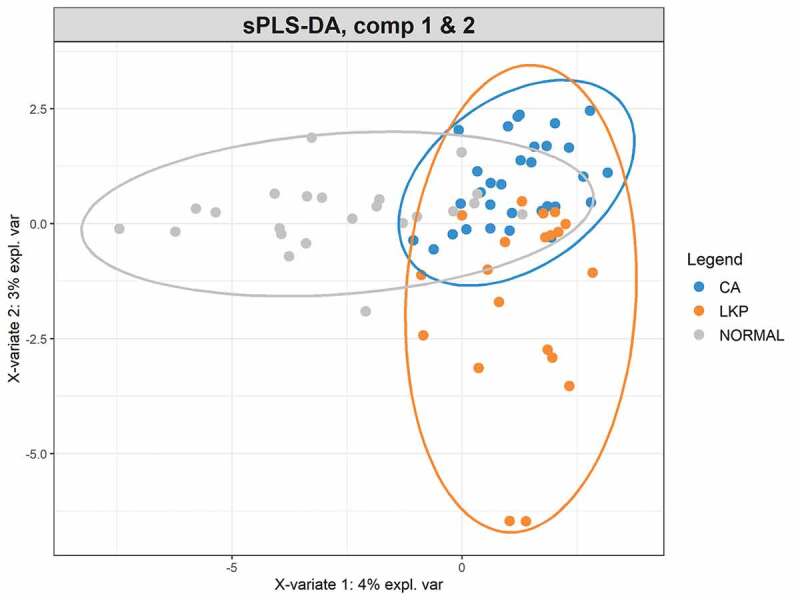
sPLS-DA plot based on the relative abundance of bacterial genera in WMF from patients in normal, LKP, and CA groups

**Figure 3.**
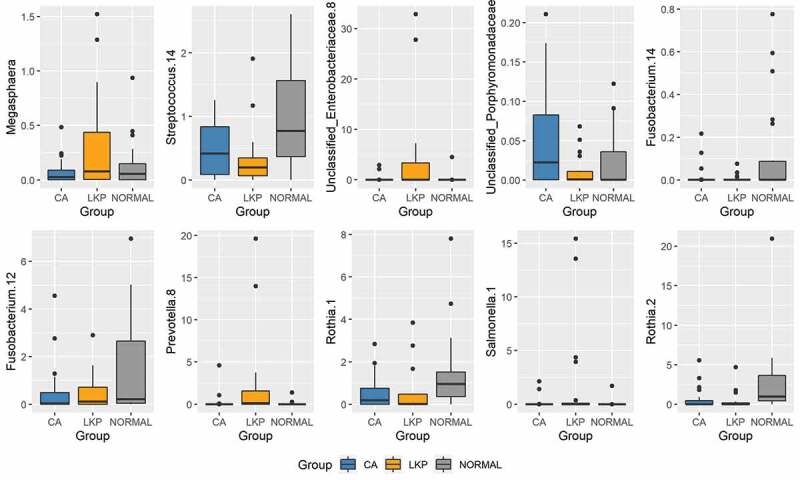
Box plots of the top 10 discriminative taxa based on the variable importance scores

A Sparse Partial Least Squares Regression Discriminant Analysis (sPLS-DA) model was built using a subset of bacterial taxa that best discriminated the studied groups. This model demonstrated a considerable overlap between the LKP and CA group whereas the majority of healthy controls were separated from both these groups on component 1 (Figure 2). CA samples formed a tight cluster; LKP could be separated from CA to a certain extent on components 1 and 2 (Figure 2). Seventy taxa were included in the first component which separated normal from the other two groups. We have illustrated the most discriminative 30 taxa to reduce the complexity (supplementary figure 1). The second component was made up of 20 taxa that mainly discriminate between the CA group and LKP group (supplementary figure 2). The relative abundances of top 10 discriminating taxa based on the variable importance scores are illustrated using the box plot (Figure 3). These were identified to belong to the following genera: *Megaspheara, unclassified enterobacteria, Prevotella, Porphyromonas, Rothia* and *Salmonella, Streptococcus, and Fusobacterium* (Figure 3).

**Figure 4.**
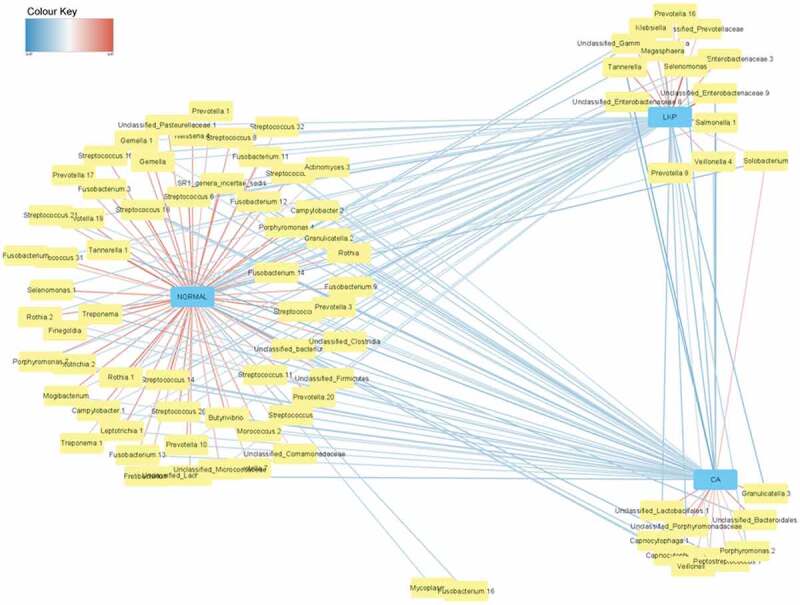
Network matrix of discriminant bacterial genera in healthy control groups identified by sparse partial least square regression (sPLS-DA). The network is displayed graphically as lines; the colour of line indicating the association level

We used network analysis that offered a predictive model to categorize the most discriminant bacterial taxa between healthy controls and LKP/CA groups; the colour of lines connecting the nodes to the centre indicates the extent of negative and positive correlations with red and blue depicting the extremes (Figure 4). Most of these taxa in healthy controls belonged to the genus *Streptococcus* followed by *Rothia* and *Fusobacterium*. The genera *Megasphaera, unclassified Enterobacteria*, and *Salmonella* and *Prevotella* were positively correlated with LKP group and *Porphyromonas* with CA groups.

### Accuracy of the sPLS-DA model

The sPLS-DA model was accurate in separating healthy controls from CA and LKP. However, due to high similarity in bacterial composition, the model has a low resolution to separate between CA and LKP (supplementary figure 3).

## Discussion

In this study, we utilized 16S rRNA metagenomics on 74 WMF samples from cohorts in the Indian subcontinent to elucidate the differences in the bacterial community dynamics in patients with oral cancer and leukoplakia patients with epithelial dysplasia relative to healthy controls. Our results illustrate that there is a shift in bacterial communities in WMF of leukoplakia and oral cancer patients which highlights the possible biomarker attributes of the bacteriome in WMF. Using a sparse PLS-DA (sPLS‐DA) derived from our current 16S metagenome analysis we demonstrated that the WMF bacterial signatures associated with leukoplakia patients with epithelial dysplasia resembled more towards oral cancer bacteriome than those in healthy controls which tend to cluster separately away from these two groups. It can be speculated that these changes may reflect the altered microenvironment and surface properties of the mucosa in diseased conditions.

With more WMF biomarker assays for clinical/commercial use in the pipeline, the collection and storage at ambient temperature are key steps to be calibrated and controlled. We have utilized a room temperature collection kit to eliminate the complexity in sample collection and storage. The use of a commercial kit ensured uniform collection in the hands of different individuals at two centres, thus reducing the disparity between samples. Stabilization of DNA in collected samples is essential for precise measurement of the bacteriome and comparison across studies. Unstabilized samples might bring in redundant variations by differential microbial growth and potential DNA degradation that may result in a bacterial snapshot which does not reflect the original community. The room temperature stabilization of DNA eliminates the requirement of −20°C/−80°C freezers for immediate storage of WMF as well as expensive and cumbersome dry ice or cold pack shipping. Since the samples are stabilized immediately, it removes the multiple freeze-thaw cycles that are detrimental in microbiome studies. Moreover, it is also helpful for the collection of samples from geographic locations where access to these storage conditions does not exist.

At the compositional level, LKP patients exhibited a decrease in *Firmicutes* and an increase in *Bacteroidetes*. This is consistent with the findings reported by Hu et al. on the salivary microbiome in Chinese population [19] and Amer et al. who reported lower levels of Firmicutes and higher levels of Bacteroidetes in oral swabs from Leukoplakia patients relative to healthy controls [20]. However, these major compositional differences at the phylum level tend to revert in oral cancer patients in our study and thus may depict a transient imbalance during LKP. We also observed a decrease in the abundance of *Actinobacteria* in WMF in LKP and oral cancer patients, relative to controls. This has been reported previously in precancer and oral cancer swabs by Schmidt et al. and Wang et al. in oral cancer tissues [21,22]. Members of this phylum may be outcompeted by commensal bacteria that have rapid growth at the relatively acidic and hypoxic tumor environment [22]. However, these studies have reported swab and tissue samples directly from the tumor site, whereas our samples represent a more generalized oral microbiome. The similarity in our data to the afore-mentioned studies may suggest that WMF may reflect the microbial composition of tumor surface. However, further studies are required that compare the salivary samples and tissue samples from the same patients to elucidate their association.

The majority of discriminant bacteria from the WMF of healthy controls relative to diseased groups belonged to the genus *Streptococcus*; which is predictable as members of this genus account for the majority of bacterial species in the oral cavity of healthy individuals [23]. The most significant discriminating genera between the LKP and oral cancer groups belonged to the following genera: *Megaspheara, unclassified Enterobacteria, Prevotella, Porphyromonas, Granulicatella, and Salmonella*. The increased presence of members of genus *Granulicatella* [24–26] and *Porphyromonas* [27,28] in oral cancer group is consistent with previous reports. More importantly, our data demonstrate that the tetrad of *Salmonella, unclassified Enterobacteriae, Prevotella and Megasphaera* were discriminative for LKP group and *Megasphaera* was abundant only in LKP group. Certain bacteria belonging to genus *Salmonella*, being facultative anaerobes, have known ability to colonize hypoxic tumours. An effector protein AvrA secreted by *Salmonella* into host cells has been involved in activation of host β-catenin signalling as well as host AKT and ERK pathways. These pathways are shown to be involved in the transformation of naive cells, i.e. to promote carcinogenesis in mice [29,30].

Some members belonging to the group of *unclassified Enterobacteriae* were also distinctive of LKP. Lee et al. have also described higher levels of genus *Escherichia* that belong to the group of *Enterobacteriae* in WMF of oral ‘epithelial precursor lesions’ (dysplasia, hyperplasia, and hyperkeratosis) relative to WMF of controls [10]. The *Enterobacteria* have the largest genomes amongst oral bacteria and have corresponding metabolic flexibility compared with other resident bacteria in the oral cavity [31,32]. *Enterobacteriae* have not been so far linked to oral cancer; however, because of their large genome it is likely that other bacteria with smaller genomes have less metabolic flexibility and may be at a disadvantage to *Enterobacteriae* in an inflammatory tumour milieu; further investigations are needed to identify and characterize this relatively complex group of organisms in the oral cavity [31].

The most discriminative taxa in WMF of LKP patients belonged to the genus *Megasphaera*. Members of this genus include gram-negative coccoid-shaped obligate anaerobes who are commensals in the oral cavity and belong to phylum *Firmicutes* and class *Negativicutes*. Increase in the abundance of this group had been reported to be associated with dental caries [33] and with bacterial vaginosis [34]. Interestingly, *Megasphaera*, as well as other bacteria involved in bacterial vaginosis, have been speculated to play a role in promoting uterine cervical dysplasia and cervical intraepithelial neoplasia [30,35,36]. While none of these above-mentioned studies can establish the nature of the relationship between *Megasphaera* and dysplasia, members of this genus may exert a pro-inflammatory effect. This contention is also consistent with many studies that reported the extent and levels of inflammation histologically in oral epithelial dysplasia to be positively correlated with the progression of dysplasia [37–40]. However, further investigation is needed to establish the role of these pro-inflammatory bacteria in the progression of oral cancer. If these results are established, they suggest a new paradigm for the relationship between inflammation and dysplasia.

We did not find any difference in diversity indices for both the richness and the evenness of bacterial communities among the three groups. However, Lee et al. [10] and Guerrero-Preston et al. [41] reported a lower diversity in WMF of head and neck cancer patients when compared to controls, whereas Wolf et al. reported higher diversity in cancer patients [42]. Interestingly, another study reported higher diversity in late-stage oral cancer when compared to controls [43]. Such disparities in diversity among reported studies might be due to differences in stages of cancer included in particular studies or due to differences in sequencing depths [8]. Even though few studies have investigated the communities in WMF and oral rinses of oral cancer patients, it should be acknowledged that WMF samples represent the biome of the oral cavity as a whole and hence could be affected by periodontal or mucosal diseases, or dental caries.

Supervised analytic techniques which are suitable in handling such extremely complex and sparse data have rarely been used in microbiome data analysis until recently [44]. ‘Variable’ selection is inevitable to select relevant information and better characterization of the study groups in high throughput biological data. Using a supervised PLS-DA we illustrate that bacteriome composition in healthy controls was distinctive while oral cancer and leukoplakia patients shared a relatively similar bacteriome profile.

A limitation of this study is the modest sample size, which may be the reason for the average performance on AUROC plot. However, despite the sample numbers, the model works well in differentiating the control group from the diseased groups. A further limitation is the lack of quantitative information on other aspects of oral habitat, amongst which periodontal status is likely to be a critical confounder [45]. Our data refer only to two populations from the Indian subcontinent, in which the incidence of both oral leukoplakia and oral cancer is amongst the highest in the world, predominantly due to smokeless tobacco and areca nut habits: the nature of the microbiome may be substantially different in Western populations in which heavy use of alcohol, smoked tobacco, and human papillomavirus infections play a larger aetiological role.

## Conclusion

Oral cancer is often considered a complex disease, consequential to an interdependent series of host–environment interactions. Thus, it is highly unlikely that oral cancer can be detected with high specificity and sensitivity with a single biomarker. Our study confirms that a dysbiotic WMF bacteriome is associated with oral leukoplakia and that this resembles oral cancer more than normal healthy controls. Compositional shifts of WMF bacteriome may, therefore, be valuable in predicting the risk of malignant transformation. Longitudinal studies would be essential to test such a hypothesis.

## Supplementary Material

Supplemental Material
