# Supplementary material for: Salivary bacterial shifts in oral leukoplakia resemble the dysbiotic oral cancer bacteriome
Source: J Oral Microbiol. 2020 Dec 9;13(1):1857998. doi: 10.1080/20002297.2020.1857998 (PMC7734041; doi:10.1080/20002297.2020.1857998)
Supplement: Supplemental Material [file ZJOM_A_1857998_SM8998.docx]

**APPENDIX**

**Supplementary Figure 1:** Graph indicating genera contributing to component 1 of the sPLS-DA plot that discriminate the healthy controls from the other two groups (LKP & CA)

**Supplementary Figure 2:** Graph illustrating genera contributing to component 2 of the sPLS-DA plot that discriminate LKP group from CA group

**Supplementary figure 3:** AUROC curve indicating sensitivity and specificity of our sPLS-DA model

**Supplementary Figure 1**


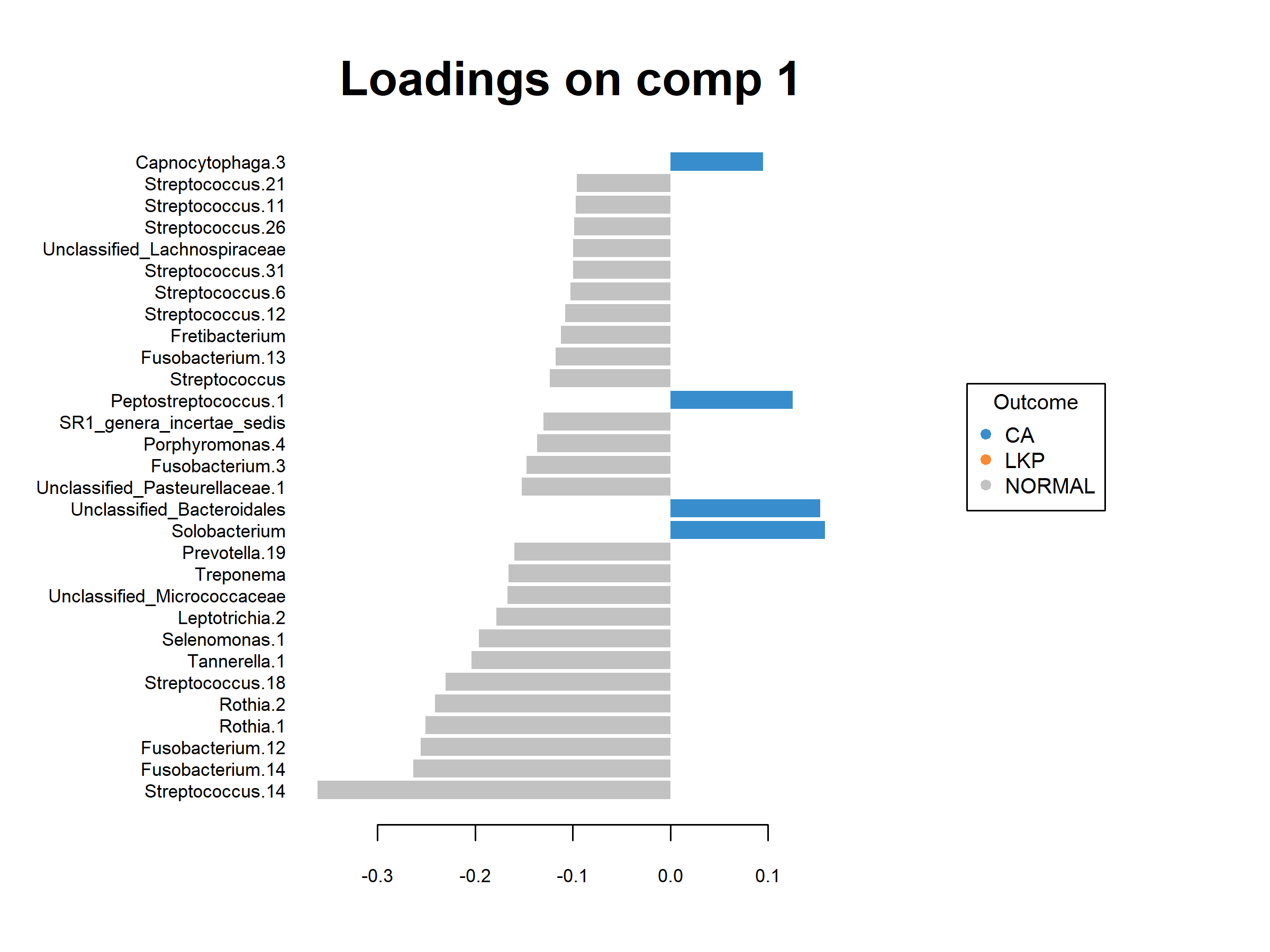


**Supplementary Figure 2**


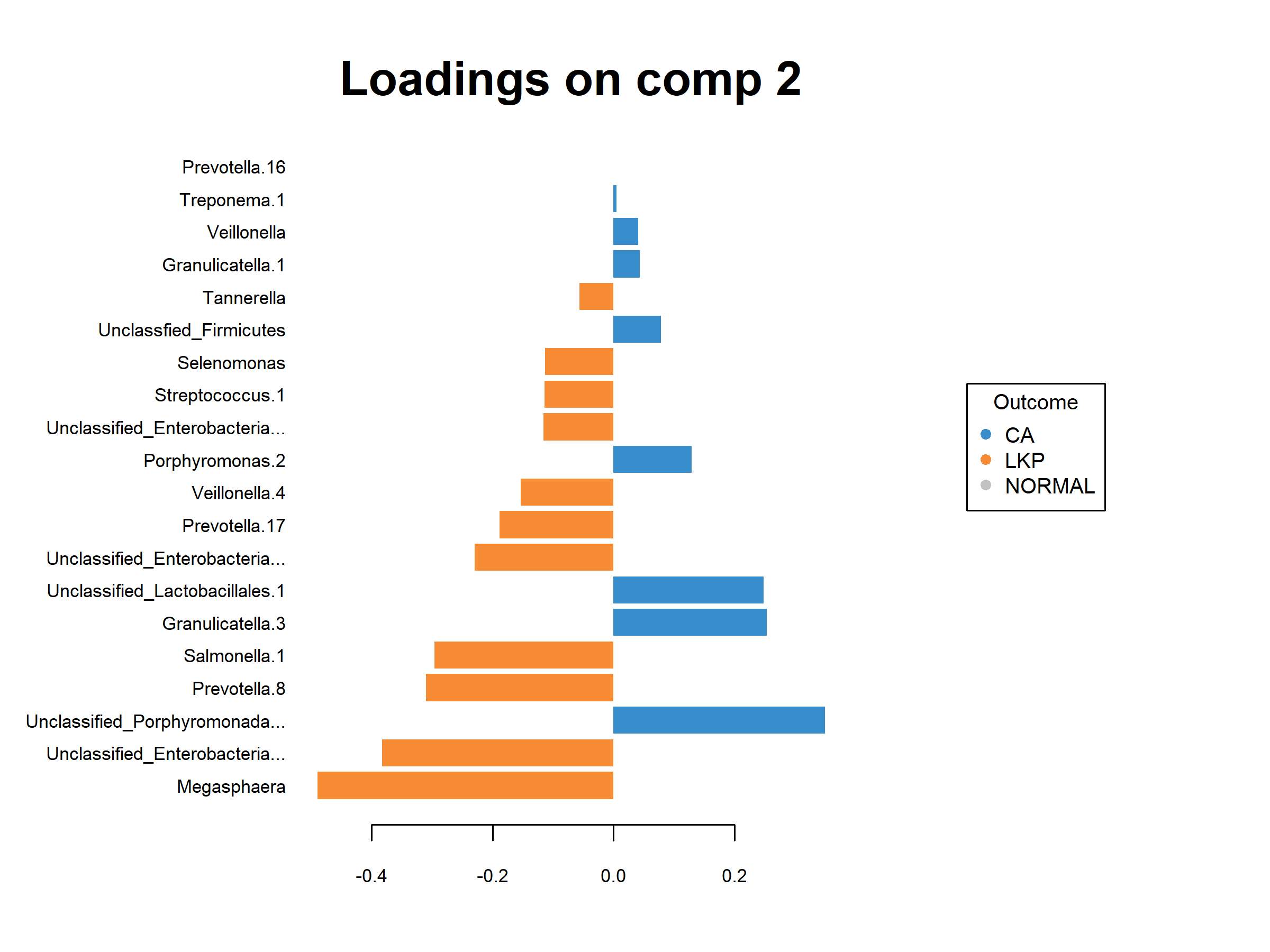


**Supplementary Figure 3**


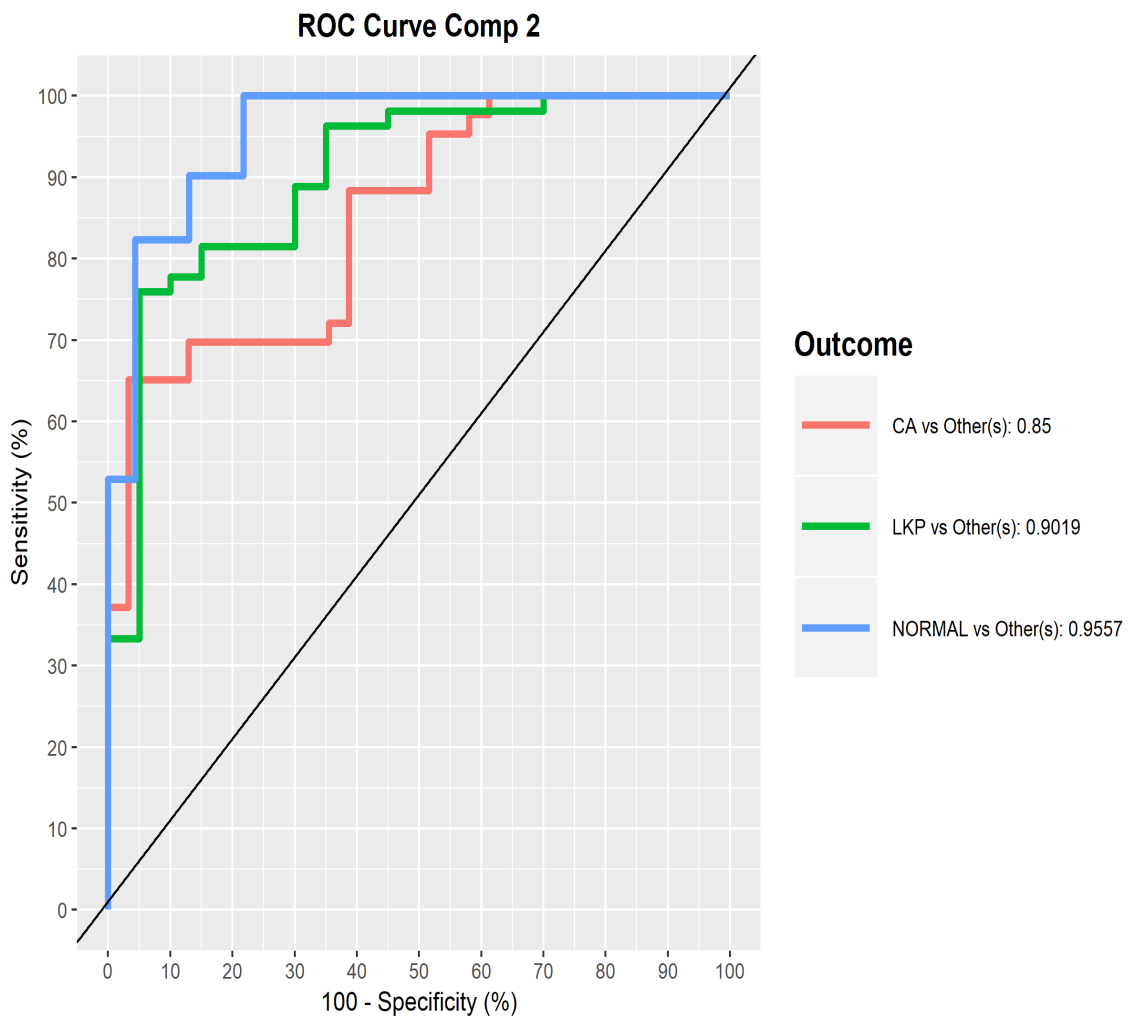


| **Parameter** | **OSCC** | **LKP** | **Normal controls** | **P value** |
| --- | --- | --- | --- | --- |
| **Total no of subjects** | 30 | 20 | 23 |  |
| **Age, mean (years)** | 49.31 | 45.67 | 43.67 | <0.05 |
| Standard deviation SD | ±13.24 | ±6.81 | ±6.81 |  |
| **Tobacco habits** |  |  |  |  |
| Smoking | 6 | 8 | 6 |  |
| Chewing | 15 | 10 | 6 | <0.05 |
| Smoking + Chewing | 10 | 2 | 3 |  |
| No habits |  |  | 8 |  |
| **Alcohol Habit** |  |  |  |  |
| Yes | 23 | 14 | 13 |  |
| No | 8 | 6 | 10 | <0.05 |
| **Anatomic sites** |  |  | NA |  |
| Floor of mouth | 3 | 0 |  |  |
| Buccal Mucosa | 12 | 11 |  |  |
| Lateral Border of tongue | 9 | 9 |  |  |
| Dorsal tongue | 6 | 0 |  |  |
| Gingiva | 1 | 0 |  |  |
| **Clinical Staging** |  | NA | NA |  |
| Lymph node + | 22 |  |  |  |
| Lymph node – | 9 |  |  |  |
| **Histological Grade of Oral CA** |  | NA | NA |  |
| Well | 11 |  |  |  |
| Moderate | 13 |  |  |  |
| Poor  **Histological Grade of LKP**  Moderate  Severe | 7  NA | 13  7 |  |  |

Supplementary Table 1: Clinical demographics of subjects
